# Mechanical Properties and Reinforcement of Paper Sheets Composited with Carboxymethyl Cellulose

**DOI:** 10.3390/polym16010080

**Published:** 2023-12-26

**Authors:** Junya Kobayashi, Masahiro Kaneko, Chamaiporn Supachettapun, Kenji Takada, Tatsuo Kaneko, Joon Yang Kim, Minori Ishida, Mika Kawai, Tetsu Mitsumata

**Affiliations:** 1 Graduate School of Science and Technology, Niigata University, Niigata 950-2181, Japan; 2 Graduate School of Advanced Science and Technology, Japan Advanced Institute of Science and Technology, Nomi 923-1292, Japan; 3 School of Chemical and Material Engineering, Jiangnan University, Wuxi 214266, China; 4 Graduate School of Modern Society and Culture, Niigata University, Niigata 950-2181, Japan

**Keywords:** paper sheet, cellulose, mechanical properties

## Abstract

The mechanical properties for paper sheets composited with glucose (Glc), methyl cellulose (MC), and carboxymethyl cellulose (CMC) were investigated. The paper composites were prepared by immersing paper sheets in aqueous solutions of these materials and drying at 100 °C for 30 min. The stress–strain curves for these paper composites were measured by a uniaxial tensile apparatus with a stretching speed of 2 mm/min. The breaking stress and strain for untreated paper were 24 MPa and 0.016, respectively. The paper composites demonstrated stress–strain curves similar to the untreated paper; however, the breaking point largely differed for these composites. The breaking strain and breaking stress for the Glc composite slightly decreased and those for the MC composite gradually increased with the concentration of materials composited. Significant increases in the mechanical properties were observed for the CMC composite. The breaking stress, breaking strain, and breaking energy for the 3 wt.% CMC composite were 2.0-, 3.9-, and 8.0-fold higher than those for untreated paper, respectively. SEM photographs indicated that the CMC penetrated into the inner part of the paper. These results strongly suggest that the mechanical improvement for CMC composites can be understood as an enhancement of the bond strength between the paper fibrils by CMC, which acts as a bonding agent. It was also revealed that the breaking strain, breaking stress, and breaking energy for the CMC composites were at maximum at the first cycle and decreased gradually as the immersion cycles increased.

## 1. Introduction

Paper sheets are obtained by casting plant fibers dispersed in water and have been widely used in our daily lives since BC. Objects for writing and drawing, clothing, and uses for wiping off dirt are typical examples. In all of these examples, the advantages of paper, being lightweight and soft, are utilized. Recently, industrial applications of paper to heavy objects have been developing rapidly. Examples include house walls, lightweight and easily buildable disaster shelters, and car bodies. At the same time, academic research is also active to improve the mechanical strength of paper so that it can be applied to much heavier objects. Actually, it has been reported that the mechanical strength of single wood pulp fibers is 90 GPa [1], and the crystal modulus distributes in a range of 138 GPa [2], surpassing those of glass fibers or aluminum.

In this paper, we focus on the mechanical property of a paper sheet and explore a facile method to improve the mechanical property, especially the break point, of a paper sheet. This is because a paper sheet often tears when an adhesive object is pealed from the paper sheet. We have studied, so far, the adhesion property between a celluloid picture and a paper sheet for the preservation of animated films [3,4]. Celluloid pictures are sheets of cellulose acetate on which manga are painted with acrylic paints, and the preservation paper is the same as general copy paper. Many of the animations were produced in the era of 1950 to 2000, and these celluloid pictures have been stored for many years until now [5,6,7,8,9]. During this storage process, the acrylic paint on the celluloid painting and the paper sheet adhered to each other. Because of the high artistic value of manga, it is very meaningful to remove the paper sheets cleanly from the manga. In our previous studies, we found that most of the preservation paper can be removed from manga when the preservation paper is wet. However, the mechanical strength of the paper also decreases, resulting in the paper remaining on the manga. To solve this problem, it is necessary to improve the mechanical strength of the paper, especially its breaking characteristics. As a first step, this study will find a simple way to improve the mechanical strength of paper in the dry state and determine the extent of the improvement.

So far, a variety of scientific studies have been conducted to improve the mechanical strength of paper sheets [10,11,12,13,14,15,16,17,18,19,20,21,22]. For example, a composite of paper and regenerated cellulose using ionic liquid has a breaking strength of approx. 100 MPa, which is approximately 1.6-fold higher than that of untreated paper [23]. It was also illustrated that a little sheet of the composite paper can lift up a heavy steel block. The composite of paper sheet and cellulose nanocrystals has a breaking strength of approx. 3.6 MPa, which is approx. 1.2-fold higher than that of untreated paper [24]. The breaking strength is not improved extensively by hybridizing cellulose nanofiber (CNFs) or nanocrystal; however, the breaking strain is greatly improved. Furthermore, the composite of paper sheets and Mxene with a breaking strength of 25.7 MPa, which is ~1.6-fold higher than that of untreated paper (16.2 MPa), has been developed for electromagnetic interference (EMI) shielding applications [25]. The enhanced mechanical property is explained as being due to a strong hydrogen–bonding interaction between Mxene sheets and the cellulose substrate [26]. In recent years, CNFs have been widely used in composite materials to improve mechanical properties [27,28,29]. For example, it has been reported that the addition of cellulose nanofiber (fiber length 60–70 μm) significantly improves the stress of paper sheets, which is approximately twice higher than without CNF [30]. The improvement of mechanical property is explained as being due to a large number of hydrogen bonds between CNF and pulp fibers. Thus, the improvement of the mechanical strength of paper sheets has been vigorously attempted with the addition of various cellulose–related substances. The procedure for these composites mentioned here is basically just dipping the paper sheet into an aqueous polymer solution, which is a simple and low–cost method that can be applied to a variety of applications. However, the optimal material and optimal concentration to improve the mechanical properties of paper sheets are not yet clear. In this study, we investigated the optimal conditions to improve the mechanical properties of paper sheets using three cellulose–related materials that have similar chemical structures to paper. The dip–dry method mentioned above was used in this study, which is simple and has significant advantages in application. In this experiment, general commercially available paper was used, not special paper; therefore, the experimental results obtained here can be applied widely.

In this paper, we investigated a methodology to improve the mechanical property of a paper sheet in a dry state, as a first step, by compositing glucose, methyl cellulose, and carboxymethyl cellulose with it. These cellulose–related materials used in this study are widely available, inexpensive materials and abundant biomass resources, making their practical use easy. The interaction between the hydroxyl groups of cellulose, which is the main component of paper, and those of the cellulose–related materials is anticipated to increase the strength of the paper sheet. The mechanical property and the reinforcement of paper sheets composited with the cellulose–related materials are presented and the physicochemical interaction between paper fibrils and these materials is discussed.

## 2. Experimental Procedures

### 2.1. Preparation of Paper Sheet Composites

A commercially available paper (R100, Japan Pulp & Paper Co., Ltd., Chuo-ku, Japan) with an apparent density of 0.78 g/cm^3^ and a tensile index of 31.9 Nm/g was provided for the mechanical test in this study. This paper also contains inorganic compounds such as calcium carbonate and silicon aluminum. Samples of the paper were immersed in aqueous solutions of glucose (Glc, Fujifilm Wako Chemicals, Osaka, Japan), methylcellulose (MC, Fujifilm Wako Chemicals, Osaka, Japan), or sodium carboxymethyl cellulose (CMC, Fujifilm Wako Chemicals, Osaka, Japan) for 60 min at room temperature. The degrees of polymerization for MC and CMC are 740 and approximately 500, respectively. The concentrations of these aqueous solutions were varied from 1 to 3 wt.%. Then, the samples were dried on a hotplate (Ninos ND–3 AS ONE Co., Osaka, Japan) at 100 °C for 30 min. For cycle experiment, the papers were wetted in the aqueous solutions and were dried by the similar manner mentioned above; this procedure was repeated for up to three cycles. The apparent density of the samples was calculated from the weight of samples (2 × 2 cm^2^) using an electronic balance (A&D Co., Ltd., GH–200, Toshima-ku, Japan).

### 2.2. Fourier-Transformed Infrared Spectroscopy

Fourier–transformed infrared (FT–IR) spectra for untreated paper sheet and its composites of Glc, MC, and CMC were measured in a wavenumber range of 400 to 4000 cm^−1^ at room temperature using an FT–IR spectrometer (Spectrum One spectrometer, Perkin–Elmer, Waltham, MA, USA) with diamond–attenuated total reflection (ATR) accessories.

### 2.3. Mechanical Measurements

The stress–strain curves for an untreated paper sheet and its composites of Glc, MC, and CMC were measured at room temperature using a uniaxial tensile apparatus with a 500 N load cell (EZ–Test EZ–SX, Shimadzu Co., Kyoto, Japan). The sample has a dumbbell shape with a size of 4 mm wide and 26 mm long, and it was stretched with a stretching speed of 2 mm/min in the machine direction (MD), which has greater fiber orientation than the cross direction (CD) [24]. The thickness of the samples was measured by an electronic micrometer (QEM133–25 Niigata Seiki Co., Sanjo, Japan). The stress is apparent stress, which was calculated from *σ* = *F*/*S*; *F* is the load and *S* is the area of the cross–section of a paper sheet without deformation. The breaking strain and breaking stress of three different samples were evaluated, and their means and standard errors are shown in the graph. The breaking stress and strain for untreated paper and its composites were also measured using their wet samples. The wet samples were prepared by immersing the dry samples in pure water for 30 s. The sample was set in the uniaxial tensile apparatus immediately after immersion.

### 2.4. Morphological Observations

A surface of the untreated paper and its composites was observed using a scanning electron microscope (SEM, JCM–6000 Neoscope JEOL Ltd., Akishima-shi, Japan) with an accelerating voltage of 15 kV. A cross–section was also observed for only paper composites with MC that has a rough surface within the sample. Samples with a Au coating were prepared using a smart coater (Dll–29010SCTR, JEOL Ltd., Akishima-shi, Japan) with a coating time of 1 min.

## 3. Results and Discussion

Table 1 shows the weight, thickness, and apparent density for an untreated paper sheet, a paper sheet after washing with pure water, and its composites of Glc, MC, and CMC with various concentrations and various cycles. The weight of the paper sheet after washing in pure water was 6.6% less than that of the untreated paper sheet. The paper sheet used in this study contains inorganic compounds such as calcium carbonate and silicon aluminum. The large decrease in the weight is probably due to these inorganics leaking out of the paper sheets into pure water during the washing process. The weight of the paper sheets immersed in the polymer solutions was higher than that of the paper sheet after washing in pure water for all samples. Also, it can be seen that the weight increase increased with polymer concentration and with the immersion cycles. This indicates that polymer chains adhered with the paper fibrils by only immersing a paper sheet in these polymer solutions. The thickness of the paper sheets after washing with pure water increased by 9.3% over the thickness of the untreated one. It is considered that the paper sheet was swollen by water, and the paper sheet was dried and solidified while keeping its expanded state. The thickness of the Glc composite became thinner with an increase in the concentration (−13%). The thickness of the MC and CMC composites increased with increases in the concentration and immersion cycles. It is considered that the paper sheet was swollen in these solutions. At a polymer concentration of 3 wt.%, the thickness increase of CMC compared to the paper sheet after washing (10%) was higher than that of MC (5.1%), which is probably due to the high hydrophilicity of CMC. Actually, when the CMC composite was taken from the aqueous solution at the second cycle, it was clearly fragile due to the swelling; the thickness at the second cycle was 34%. The thickness at the third cycle was 48%, suggesting that the CMC composite further swelled at the third cycle.

**Figure 1:**
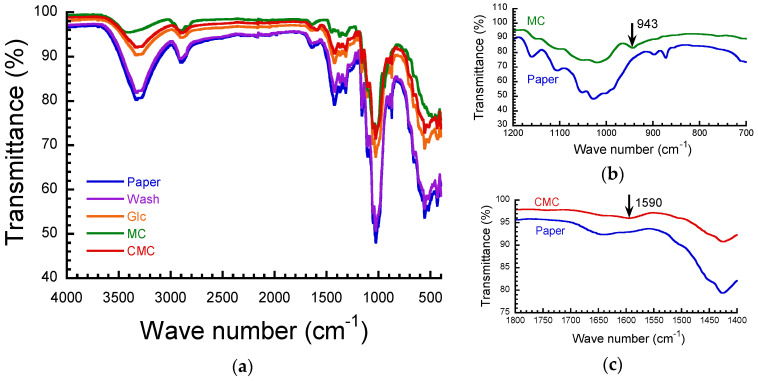
(**a**) FTIR spectra for untreated paper, paper after washing with pure water, and paper composites with Glc, MC, and CMC at a concentration of 3 wt.%. Enlarged spectra for paper composites with (**b**) MC and (**c**) CMC.

Figure 1a shows the FTIR spectra for untreated paper, paper after washing with pure water, and its paper composites with Glc, MC, and CMC at a concentration of 3 wt.%. Untreated paper exhibited strong peaks at 3340 cm^−1^ and 1030 cm^−1^, which are attributed to the stretching of the O–H bond (~3400 cm^−1^) and C–O stretching (~1000 cm^−1^), respectively. No clear difference in the wavenumber of these peaks was observed for the Glc, MC, and CMC composites. Figure 1b,c shows enlarged views of the FTIR spectra for the MC and CMC composites, respectively. The spectrum for the MC composites exhibited a peak at 943 cm^−1^, which is attributed to the C–H stretching of the CH_2_ and CH_3_ groups (~950 cm^−1^). The spectrum for CMC composites appeared with a peak at 1590 cm^−1^, which is attributed to the –COO antisymmetric stretching (~1600 cm^−1^). However, there was no clear shift in these peaks by compositing paper sheets with MC or CMC. Accordingly, it is clear that there is little interaction between the paper and these substances, although MC or CMC adhered to the paper sheets by immersing in these aqueous solutions.

**Figure 2:**
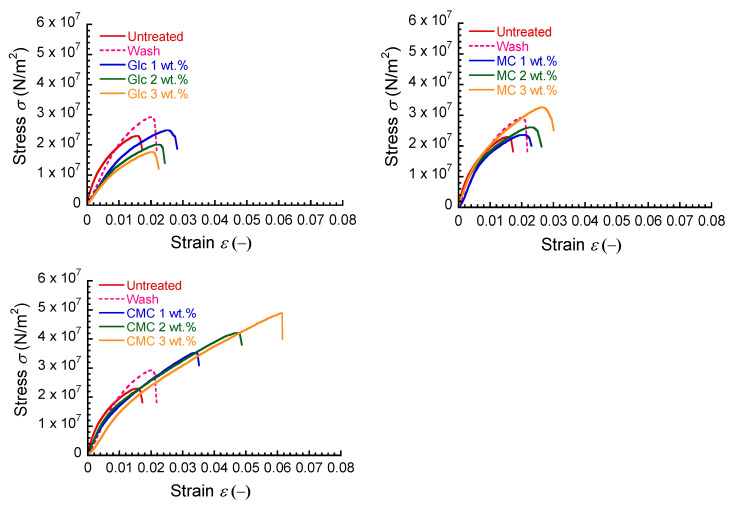
Stress–strain curves for untreated paper, paper after washing with pure water, and paper composites with Glc, MC, and CMC at various concentrations.

Figure 2 shows the stress–strain curves for untreated paper sheet, paper after washing with pure water, and paper composites with Glc, MC, and CMC at various concentrations. The stress–strain curve for the untreated paper showed a break point at a strain of approximately 0.016. The breaking stress and breaking strain for paper sheet after washing with pure water were higher than those of untreated one. The mechanical strength for the Glc composite decreased as increasing the concentration and oppositely it for the MC composite increased with the concentration. It was also observed composites that the initial slope of the stress–strain curve for Glc (~1.7 GPa), i.e., Young’s modulus, decreased remarkably compared to the untreated paper (~2.5 GPa), while that for MC remained unchanged. The mechanical strength for the CMC composites was greatly improved as increasing the concentration while almost keeping the Young’s modulus. After the break point, the Glc and MC composites showed similar stress–strain curves with that of the untreated paper without showing a sharp decrease in stress. On the other hand, the stress–strain curve of the CMC composite demonstrated a rapid decrease.

**Figure 3:**
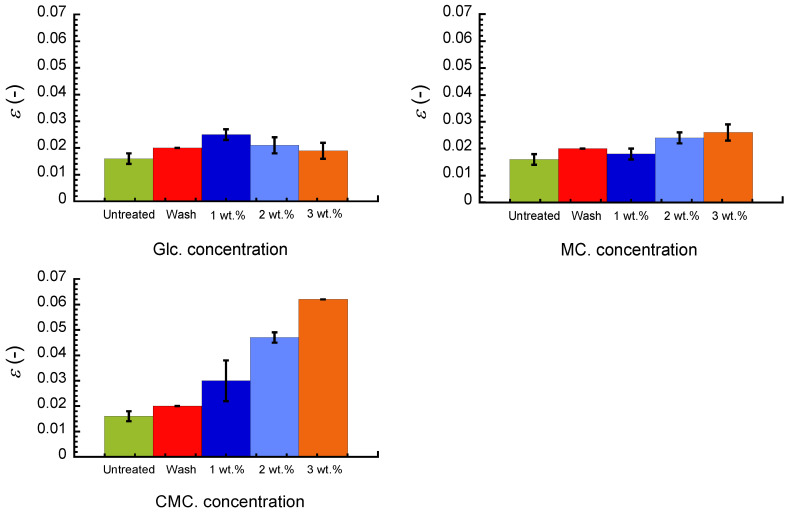
Breaking strain for untreated paper, paper after washing with pure water, and its composites of Glc, MC, and CMC with various concentrations.

Figure 3 demonstrates the breaking strain for an untreated paper sheet, paper after washing with pure water, and paper composites with Glc, MC, and CMC at various concentrations. The paper sheet after washing with pure water exhibited a slightly high value of 0.02, which was higher than that of untreated paper. The breaking strain of the Glc composite was at maximum at 1 wt.% and gradually decreased with the concentration. On the other hand, the breaking strain for the MC composite gradually increased with the concentration; the breaking strain at 3 wt.% was approximately 1.6-fold higher than that of the untreated paper. The breaking strain of the CMC composites increased dramatically with the concentration; the breaking strain at 3 wt.% was approximately 3.9-fold higher than that of the untreated paper. It is worth mentioning that the Young’s modulus for the CMC composite (~2.1 GPa) was comparable with that of the untreated paper (~2.5 GPa). This indicates that the bond strength or the amount of bonding between paper fibrils was not raised by compositing the paper with CMC. Seth and Page reported stress–strain curves of paper sheets with the addition of the bonding and debonding agents [14]. When the bonding agent of locust bean gum is composited, the adhesive strength between the paper fibers increases. As a result, the breaking strain and tensile strength of the composite paper are higher than those of the untreated paper. Young’s modulus and the apparent density of the composite paper are similar to those of untreated paper. In other words, the stress–strain curve of the composite paper with locust bean gum has the same shape as that of the untreated paper; only the endpoint is higher. On the other hand, when a debonding agent of Hyamine 2389 is composited, the adhesive strength between the paper fibers becomes lower. As a result, the breaking strain and tensile strength of the composite paper are lower than those of the untreated paper. Young’s modulus and apparent density are similar to those of the untreated paper. In other words, the stress–strain curve of the composite paper has the same shape as that of untreated paper; only the endpoint is lower. Thus, it can be understood that the shape of the stress–strain curve is not affected by the adhesive strength between the paper fibers and bonding agents. The strain–stress curve for the CMC composites observed here can be understood as an enhancement of the bond strength between paper fibrils by CMC, which acts as a bonding agent. Ruonan et al. reported similar behavior for a paper composite with regenerated cellulose using an ionic liquid from hand layup technology [23]. The slope of the stress–strain curves for untreated paper and the paper composite with 4% regenerated cellulose are nearly identical at low strains, which is similar to the results of Seth and Page mentioned above. The breaking strength and braking strain for the paper composite are 101 MPa and ~17.5%, respectively, which are 1.6- and 3.1-fold higher than those of the untreated paper. The CMC composite in this study achieved 2.1-fold higher breaking strength and 3.9-fold higher breaking strain with an addition of 3% CMC.

**Figure 4:**
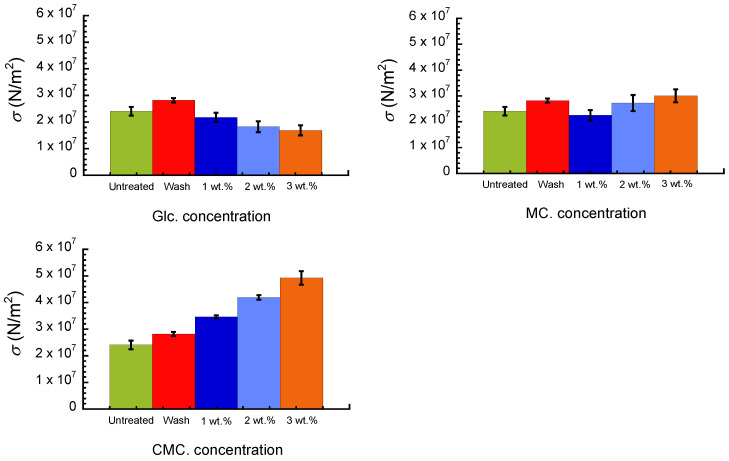
Breaking stress for untreated paper, paper after washing with pure water, and its composites with Glc, MC, and CMC with various concentrations.

Figure 4 shows the breaking stress for an untreated paper sheet, paper after washing with pure water, and paper composites with Glc, MC, and CMC at various concentrations. The paper sheet after washing with pure water exhibited a slightly high value of 28 MPa, which was higher than that of untreated paper. The breaking stress for Glc composites decreased gradually with the concentration. Oppositely, the breaking stress for the MC composite demonstrated a trend to increase with the concentration; the breaking stress at 3 wt.% was approximately 1.2-fold higher than that of the untreated paper. The breaking stress for the CMC composite increased dramatically with the concentration; the breaking stress at 3 wt.% was approximately twice compared to that of the untreated paper. The breaking stress and breaking strain for the paper sheet after washing with pure water were higher than those for untreated paper. This might be due to the interaction between paper fibers being enhanced by the leaking of these inorganic compounds during the washing process. The breaking strains for the untreated paper sheet and paper composites with Glc, MC, and CMC in the wet state were 0.010, 0.015, 0.009, and 0.003, respectively. The breaking stresses for the untreated paper sheet and paper composites with Glc, MC, and CMC in the wet state were 1.0, 0.5, 0.3, and 0.3 MPa, respectively. Thus, the mechanical enhancement is only observed for the paper composites in a dry state, and the mechanical properties were observed to be significantly reduced by wetting, particularly for the CMC composite. Clearly, wetting a paper sheet cannot be applied to a case for removing the paper sheet from paint, as explained in the Introduction. However, the swelling behavior of paper sheets coated with hydrophilic polymers such as CMC might be effective for peeling the paper from paint.

**Figure 5:**
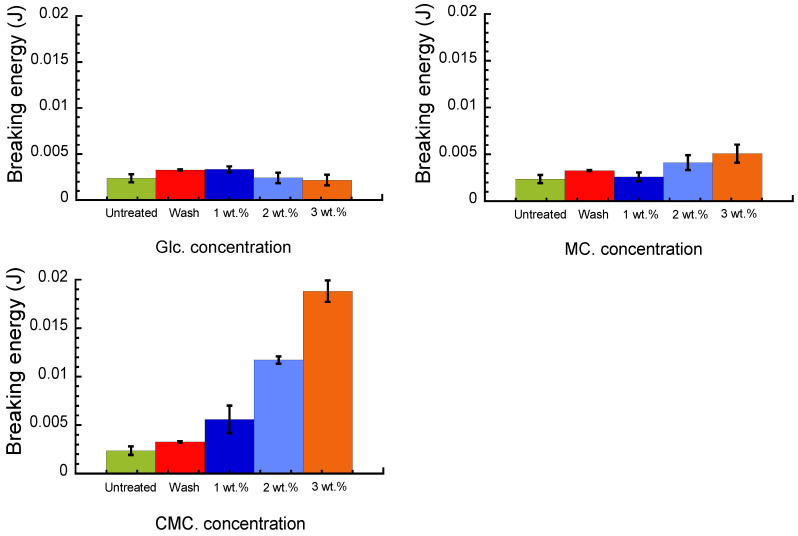
Breaking energy for untreated paper, paper after washing with pure water, and its composites with Glc, MC, and CMC with various concentrations.

Figure 5 exhibits the breaking energy for an untreated paper sheet, paper after washing with pure water, and paper composites with Glc, MC, and CMC at various concentrations. The breaking energy was calculated by integrating the stress over the region from the natural length to the breaking strain. The breaking energy for the Glc composite was almost independent of the concentration; meanwhile, the breaking energy for the MC composite increased gradually with the concentration. The breaking energy for the MC composite at 3 wt.% was approximately 2.1-fold higher than that for untreated paper. The breaking energy for the CMC composite was significantly higher at higher concentrations; the breaking energy at 3 wt.% was approximately 8.0-fold higher than that of the untreated paper. It was also found that the breaking energy for CMC composites increased in proportion to the CMC concentration. This means that the elastic energy stored in the composite is proportional to the CMC concentration. Hence, it can be explained by the increase in the elastic energy being attributed to the increase in the number of CMC chains connecting between the paper fibrils that elongate by the strain. As described in the Introduction, the reinforcing effect of the mechanical properties is explained by the amount of hydrogen bonding [26]. Ma et al. investigated the strength of hydrogen bonding in nanocomposite paper using Fourier-transform infrared (FTIR) spectra and X-ray photoelectron spectroscopy (XPS). The FTIR spectrum of the nanocomposite paper shows the appearance of a shoulder peak and a peak shift of the OH group to the low wavenumber side. The XPS of the nanocomposite paper also undergoes a shift of the bonding peak to the higher energy side. Therefore, the formation of hydrogen bonds arises between MXene and cellulose. The authors pointed out that the numerous hydrogen-bonding interactions will contribute to the enhanced interface adhesion and mechanical properties for homogeneously blended nanocomposite papers. In the present experiment, the molar concentrations of -OH groups in the aqueous solutions of Glc, MC, and CMC were calculated to be 8.3, 1.6, and 2.5 mol/L, respectively, when the weight concentration was 3.0 wt.%. Accordingly, there is no simple correlation between the number of -OH groups and the mechanical features of these paper composites, on the assumption of constant absorption of these materials into the paper.

**Figure 6:**
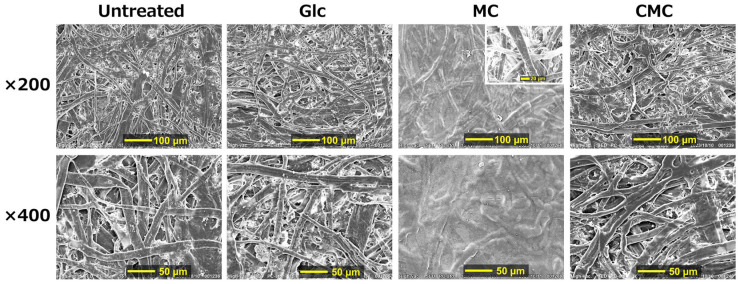
SEM photographs for untreated paper and its composites with Glc, MC, and CMC with a concentration of 3 wt.%. Inset: a cross-section of paper composites with MC.

Figure 6 displays the SEM photographs for an untreated paper sheet and paper composites with Glc, MC, and CMC at a concentration of 3 wt.%. There was no significant difference between the photographs of the Glc composites and those of the untreated ones. The photos for the MC composite were clearly unsharp compared to the other photos; however, the photo of a cross-section was clear (inset of Figure 6). These results strongly indicate that only surface topography was greatly changed to a flatter surface by compositing the paper sheet with MC. Accordingly, it is considered that the most of the MC chains are adhered to and deposited on the surface and fewer penetrate into the inside of the paper sheet. Also, the slight increases in the mechanical properties such as breaking stress and energy are considered to be caused by the MC being coated on the surface of the paper. For CMC composites, the photograph was clear similarly to those of untreated paper and Glc composites, suggesting that the CMC penetrated into the inner part of the paper.

**Figure 7:**
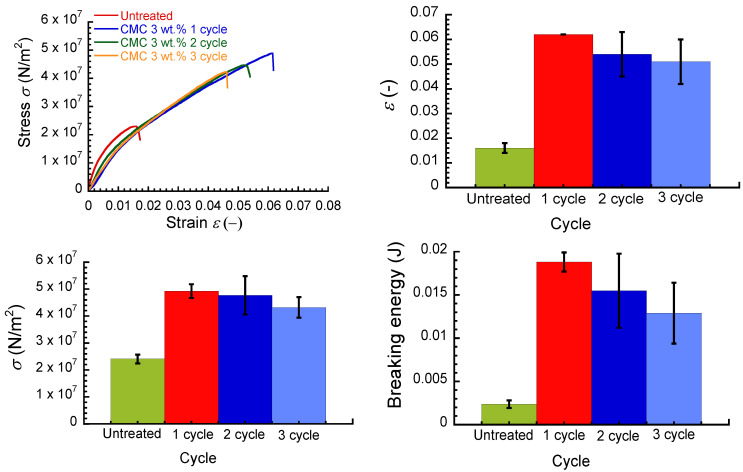
Effect of immersion cycles on the stress–strain curves, breaking strain, breaking stress, and breaking energy for untreated paper and its composites with Glc, MC, and CMC with a concentration of 3 wt.%.

Figure 7 shows the effect of immersion cycles on the strain–stress curve, breaking strain, breaking stress, and breaking energy for an untreated paper sheet and CMC paper composite at a concentration of 3 wt.%. Irrespective of the immersion cycles, the CMC composites exhibited similar stress–strain curves. The breaking strain, breaking stress, and breaking energy achieved a maximum in the first cycle and decreased gradually as the immersion cycles increased. It is very interesting that the mechanical properties of CMC composites demonstrated deterioration in the second and third cycles. This may be due to the hydrophilic property of CMC. In the second and third cycles, the CMC composite was fragile when the composite was taken from the CMC aqueous solution. This strongly indicates that the CMC composite was swollen by the aqueous solution, and the paper fibrils transformed into a sparse structure. Actually, the apparent density for the CMC composite decreased remarkably with the cycles, which supports this idea. As listed in Table 1, both weight and thickness for the CMC composites increased as the immersion cycles increased; therefore, the amount of CMC that adhered increased with the immersion cycles. The density decreased with the increasing immersion cycles due to the swelling. It is considered that the amount of adhered CMC is a dominant parameter, rather than density, for the mechanical property.

**Figure 8:**
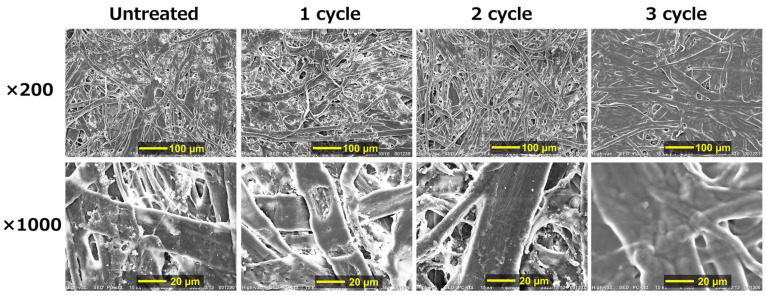
SEM photographs for paper composites with 3 wt.% CMC with various cycles of immersion process.

Figure 8 presents the SEM photographs for an untreated paper sheet and its composites with 3 wt.% CMC for which various cycles of the immersion processes were performed. It was observed that the structure of the paper fibrils was changed from dense to sparce as the immersion cycles increased, which is consistent with the result of the density. Moreover, the photo of the first cycle exhibited that the paper fibrils seemed to have a smooth surface compared to the untreated paper. Therefore, it can be considered that the CMC adhered on the paper fibrils at the first immersion, and the adhered CMC was swollen by water after the second immersion. This swelling might expand the distance between the fibers; actually, the CMC composite after the second immersion was very fragile in a wet state. After the second immersion, the CMC connecting the paper fibrils may have changed to a structure that allows slippage between the CMC chains, resulting in the reduction in the breaking stress and strain.

## 4. Conclusions

The mechanical properties for paper sheets composited with various cellulose-related materials, glucose, methyl cellulose, and carboxymethyl cellulose, were investigated. Significant increases in the mechanical properties were observed for a paper sheet composite with CMC; the breaking stress, breaking strain, and breaking energy for a paper sheet composite obtained by immersing in 3 wt.% CMC aqueous solution were 2.0-, 3.9-, and 8.0-fold higher than that for untreated paper, respectively. On the other hand, Young’s modulus for CMC composites was comparable with that for untreated paper; i.e., only the fracture behavior was changed. Furthermore, morphological studies indicate that CMC penetrated into the inner part of the paper and covered the paper fibrils. Therefore, the significant improvement in the breaking strength and breaking strain of CMC composites may be caused by the interconnection and elongation of CMC covering the paper fibrils. The results obtained in this study suggest that this method would be effective as a method to cleanly peel off adhesions such as paint from paper sheets. In addition to the problem of adhesion of acrylic paints to animation cels, we believe that it can also apply to the preservation of ancient cultural properties.

## Figures and Tables

**Table 1:** Weight, thickness, and apparent density for untreated paper sheet, paper sheet after washing with pure water, and its composites of Glc, MC, and CMC with various concentrations and various immersion cycles.

	Weight (g)				Thickness (mm)				Density (g/cm^3^) ^(a)^				
		Concentration (wt.%)				Concentration (wt.%)				Concentration (wt.%)			Cycle ^(b)^
		1	2	3		1	2	3		1	2	3	
Untreated	0.0273	–	–	–	86.3	–	–	–	0.78	–	–	–	–
Wash	0.0255	–	–	–	94.3	–	–	–	0.68	–	–	–	1
Glc	–	0.0270	0.0279	0.0316	–	96.0	93.3	88.2	–	0.68	0.73	0.81	1
MC	–	0.0262	0.0273	0.0278	–	89.8	90.9	99.1	–	0.75	0.77	0.80	1
CMC	–	0.0261	0.0265	0.0282	–	97.3	101.2	104.0	–	0.67	0.66	0.68	1
	–	–	–	0.0294	–	–	–	126.8	–	–	–	0.59	2
	–	–	–	0.0314	–	–	–	139.8	–	–	–	0.57	3

^(a)^ Apparent, ^(b)^ Immersion cycle.

## Data Availability

Data are contained within the article.
